# Editorial: Endocrine and metabolic consequences of childhood obesity, volume III

**DOI:** 10.3389/fendo.2025.1546470

**Published:** 2025-03-24

**Authors:** Elpis Vlachopapadopoulou, Artur Mazur, Aneta Monika Gawlik, Grzegorz Telega, Malgorzata Wojcik, Dénes Molnár

**Affiliations:** ^1^ Department of Endocrinology-Growth and Development Panagiotis, Aglaia Kyriakou Children’s Hospital, Athens, Greece; ^2^ Department of Pediatrics, Childhood Endocrinology and Diabetes, Medical College of Rzeszow University, Rzeszow, Poland; ^3^ Department of Pediatrics and Pediatric Endocrinology, Faculty of Medical Sciences, Medical University of Silesia, Katowice, Poland; ^4^ Department of Pediatrics, Medical College of Wisconsin, Milwaukee, WI, United States; ^5^ Department of Pediatric and Adolescent Endocrinology, Chair of Pediatrics, Pediatric Institute, Jagiellonian University Medical College, Krakow, Poland; ^6^ Department of Pediatrics, Medical School, University of Pécs, Pécs, Hungary; ^7^ National Laboratory for Human Reproduction, Szentágothai Research Centre, University of Pécs, Pécs, Hungary

**Keywords:** children, obesity, insulin resistance, sarcopenia, asprosin, zonulin, gut microbiota, irisin

Childhood obesity has been recognized by the World Health Organization as the most important public health issue in developed countries (1).[WHO]. Previously, obesity was primarily viewed as the result of personal choices and lifestyle factors. However, this perspective has shifted, and it is now recognized as a chronic, relapsing disease that is challenging to treat. Obesity involves complex genetic, physiological, socioeconomic, and environmental factors and is associated with numerous early and late comorbidities and disabilities (2) Key factors contributing to increased adiposity include genetic and epigenetic influences that act even before conception, during pregnancy, and in the postnatal period, along with environmental and social factors that shape food preferences and physical activity choices (3) The need to address people-first language in childhood overweight and obesity and to address weight bias and stigma has been recognized and advocated by a consortium of Scientific Societies (4). The American Academy of Pediatrics has published new guidelines that highlight the importance of early recognition of contributing factors and children at higher risk for overweight or obesity and call for early action (2).

Furthermore, it is very important, from a scientific point of view, to investigate new molecules, and new pathways associated with obesity in addition to mechanisms that contribute to the obesity phenotype and its comorbidities, and to investigate novel ways to address them. Thus, this is the third Research Topic on Endocrine and Metabolic Comorbidities of childhood obesity.

In this Research Topic, researchers have investigated associations of recently described proteins such as irisin, zonulin (5), asprosin (6) and angiopoietin-like protein 8 (ANGPTL8) (7) with anthropometric and metabolic parameters, while others have reported on the importance of gut microbiota, and inflammatory Th17 cells in regard to sleep apnea and insulin resistance. An association between increased ANGPTL8 and increased body weight was reported, along with the impact of maternal diet during pregnancy on the development of obesity in the offspring. The effect of novel treatment options such as exenatide on secretory patterns of endogenous hormones was also highlighted. The importance of recognizing the presence of sarcopenia was documented. The association of the two novel composite inflammatory markers: The Systemic Immune-Inflammatory Index (SII) and the Systemic Inflammatory Response Index (SIRI) with body mass index (BMI) in children was first reported based on epidemiological data.

More specifically, the studies included here address physiology issues, inflammation, epigenetics and therapeutic intervention. Important findings include the following:

ANGPTL8 levels were found to be elevated in adolescents with overweight and obesity, in the Arab population of Kuwait, with a strong positive correlation with hsCRP, leptin, and chemerin. This study is important since ANGPTL8 has diverse biological effects depending on its location and is involved in various pathological processes—such as inflammation, tumor progression, ventricular remodeling, and ectopic fat deposition—elevated levels during early adolescence could serve as an early marker for metabolic and cardiovascular complications and a potential target for novel pharmacological interventions.

Zonulin is a protein secreted mainly by the liver and the enterocytes and it represents the only measurable blood protein known to regulate the permeability of intestinal tight junctions. Furthermore, it may play a role in the pathogenesis of obesity and related metabolic disturbances. The first documentation of the meal-related secretion pattern of serum zonulin in a pediatric cohort suggests that hyperglycemia during an OGTT or post-meal in real-life conditions may lead to prolonged upregulation of zonulin, potentially impacting intestinal function. This finding is significant, as it highlights the connection between intestinal permeability, inflammation, and obesity, reinforcing the rationale for developing novel therapeutic strategies targeting microbiome modifications.

The correlation found between HOMA-IR and plasma asprosin levels (a fasting-induced protein hormone that regulates hepatic glucose release, obesity, and insulin resistance) in Korean children and adolescents in a clinical setting was found to have clinical significance in the pathophysiology of fatty liver.

Irisin, a recently identified adipomyokine, exerts a range of effects that include influences on metabolism, energy balance, insulin resistance, and the browning of white adipose tissue. In a study published in this Research Topic, irisin levels were found to correlate positively with fasting glucose, insulin, HOMA-IR, and osteocalcin, while showing a negative correlation with HDL. Additionally, the research demonstrated a positive association between irisin levels and total body-less head (TBLH) BMD z-score in Korean children and adolescents, highlighting that irisin is a significant mediator for metabolic actions.

Currently, there is no consensus on diagnostic criteria for pediatric sarcopenic obesity. In this Research Topic, one study assessed obese children and adolescents for muscle mass, muscle strength and physical performance, reporting that sarcopenia detection varied with the criteria used. Further research using gold standard measures of body composition is required, specific to age and pubertal stages, with robust normative data.

Age- and puberty- appropriate measures that identify insulin resistance may be useful in the assessment and treatment of pediatric and adolescent obesity, going beyond the flawed assessment based on fasting insulin levels alone. In this Research Topic, a surrogate insulin-resistant estimate, SPISE (Single Point Insulin Sensitivity Estimator), was examined through puberty. SPISE requires fasting measures of HDL-cholesterol and triglycerides and the measurement of body mass index. SPISE values were significantly lower in patients with confirmed insulin resistance (total sum of insulin OGTT ≥ 535 μu/mL) in all pubertal groups. The authors concluded that the strength of SPISE lies in the use of widely accessible and affordable laboratory tests, making it well-suited for large-scale studies and ongoing monitoring across various populations.

A narrative review explored the literature on human studies of how maternal intake of macronutrients and vitamins affects DNA methylation patterns and their link with offspring phenotypes, such as obesity and metabolic changes. The authors emphasize the increasing evidence that maternal consumption of specific nutrients—such as fructose, fat, protein, vitamins, and methyl-group donors—during pregnancy may alter DNA methylation patterns in the offspring, potentially contributing to obesity and related metabolic disorders.

Trying to elucidate the connection between increased adiposity and low-grade inflammation in the body, the association between erythrocyte parameters, the proinflammatory Th17 lymphocytes, and IR markers was evaluated in children with excessive body weight. The study published in this Research Topic confirmed that erythrocyte parameters such as erythrocyte count and HGB concentration are elevated in children with obesity and show a positive correlation with insulin resistance and proinflammatory Th17 lymphocytes. The clinical relevance of measuring these parameters lies in their role as indirect markers of low-grade systemic inflammation that can be used to evaluate the impact of obesity in individuals and monitor their response to treatment, whether through diet and exercise or pharmacological intervention.

The only article addressing treatment effects is the collaborative study between the Upsala, Sweden and Salzburg, Austria groups that evaluated the effect of exenatide extended-release treatment in adolescents with obesity in a randomized, controlled manner. The authors have previously published findings on BMI, cholesterol and glucose levels along with the safety and tolerability of the medication. Exenatide was found to reduce BMI-SDS, weight, waist circumference, 2-hour glucose during OGTT, and total cholesterol, without significant changes in liver fat content in comparison to placebo. The safety and tolerability profiles were comparable to placebo with the exception that mild adverse events were more frequent in exenatide-treated patients. (Weghuber) In this sub-study the authors evaluated before and after treatment values of GLP-1, glucose, insulin, glucagon and increased glicentin levels that were measured during OGTT and DPP-4 and proinsulin that were measured at fasting The authors concluded that treatment with weekly subcutaneous injections of 2 mg of exenatide extended-release did not affect GLP-1 levels during OGTT. Treatment significantly lowered DPP-4, proinsulin and the proinsulin-to-insulin ratio at fasting, increased glicentin levels but did not affect insulin, C-peptide or glucagon levels during OGTT.

By maintaining total GLP-1 levels and reducing DPP-4 (which degrades GLP-1), exenatide prolongs the effects of the hormone’s (GLP-1) leading to improved glucose homeostasis, by enhancing glucose-dependent insulin secretion and decreasing hepatic glucose production.

In conclusion, the studies featured in this Research Topic shed light on various pathways involved in the development of obesity and explore potential associations between different biomarkers, anthropometric measurements, and metabolic parameters, with a special focus on insulin resistance. Additionally, they raise important scientific questions for future research on the etiology and metabolic complications of childhood obesity.
